# New Altered Non-Fibrillar Collagens in Human Dilated Cardiomyopathy: Role in the Remodeling Process

**DOI:** 10.1371/journal.pone.0168130

**Published:** 2016-12-09

**Authors:** Carolina Gil-Cayuela, Esther Roselló-LLetí, Ana Ortega, Estefanía Tarazón, Juan Carlos Triviño, Luis Martínez-Dolz, José Ramón González-Juanatey, Francisca Lago, Manuel Portolés, Miguel Rivera

**Affiliations:** 1 Cardiocirculatory Unit, Health Institute of La Fe University Hospital (IIS La Fe), Valencia, Spain; 2 Genomic Systems, Paterna (Valencia), Spain; 3 Heart Failure and Transplantation Unit, Cardiology Department, La Fe University Hospital, Valencia, Spain; 4 Cellular and Molecular Cardiology Research Unit, Department of Cardiology and Institute of Biomedical Research, University Clinical Hospital, Santiago de Compostela, Spain; University of California Los Angeles David Geffen School of Medicine, UNITED STATES

## Abstract

**Background:**

In dilated cardiomyopathy (DCM), cardiac failure is accompanied by profound alterations of extracellular matrix associated with the progression of cardiac dilation and left ventricular (LV) dysfunction. Recently, we reported alterations of non-fibrillar collagen expression in ischemic cardiomyopathy linked to fibrosis and cardiac remodeling. We suspect that expression changes in genes coding for non-fibrillar collagens may have a potential role in DCM development.

**Objectives:**

This study sought to analyze changes in the expression profile of non-fibrillar collagen genes in patients with DCM and to examine relationships between cardiac remodeling parameters and the expression levels of these genes.

**Methods and Results:**

Twenty-three human left ventricle tissue samples were obtained from DCM patients (n = 13) undergoing heart transplantation and control donors (n = 10) for RNA sequencing analysis. We found increased mRNA levels of six non-fibrillar collagen genes, such as *COL4A5*, *COL9A1*, *COL21A1*, and *COL23A1* (*P <* 0.05 for all), not previously described in DCM. Protein levels of *COL8A1* and *COL16A1* (*P* < 0.05 for both), were correspondingly increased. We also identified TGF-β1 significantly upregulated and related to both COL8A1 and COL16A1. Interestingly, we found a significant relationship between LV mass index and the gene expression level of *COL8A1* (r = 0.653, *P* < 0.05).

**Conclusions:**

In our research, we identified new non-fibrillar collagens with altered expression in DCM, being *COL8A1* overexpression directly related to LV mass index, suggesting that they may be involved in the progression of cardiac dilation and remodeling.

## Introduction

Dilated cardiomyopathy (DCM) is one of the most common types of cardiac diseases and is clinically characterized by ventricular chamber dilation and systolic dysfunction that commonly results in ventricular arrhythmias and heart failure (HF) [1]. Although this syndrome is a common reason for cardiac transplantation in adults and children, its etiology remains unknown [1,2]. In DCM, cardiac failure is accompanied by profound alterations of extracellular matrix (ECM) architecture [3], including changes in collagen concentration and cross-linking. Although new collagen is deposited in an effort to reinforce the ventricle wall, increased activity of matrix metalloproteinases (MMPs) and collagen turnover lead to a deficiency in cross-linking and augmented ECM compliance. This weakening of structural linkages is accompanied by myocyte lengthening, promoting the progression of cardiac dilation, and left ventricular (LV) dysfunction [4,5].

The ECM consists predominantly of collagens, as well as proteoglycans, glycosaminoglycans, adhesion proteins, and signaling molecules [6,7]. Collagens contribute to the mechanical properties, organization, and morphology of tissues. They also regulate cell adhesion, proliferation, migration, differentiation, and apoptosis processes, in which transforming growth factor beta-1 (TGF-β1) usually plays a central role in the propagation of intracellular signaling [7–9].

In the diseased heart, fibrillar collagens are more abundant than non-fibrillar collagens despite the latter consisting of more classes [10]. Major classes of non-fibrillar collagens include network-forming collagens, fibril-associated collagens with interrupted triple helices (FACITs), membrane-associated collagens with interrupted triple helices (MACITs), and multiple triple-helix domains and interruptions (MULTIPLEXINs) [10,11]. Although in cardiomyopathies non-fibrillar collagens are less abundant than fibrillar collagens, this does not necessarily entail a lower functional relevance [12,13]. In fact, we recently found overexpression of non-fibrillar collagens in ischemic hearts, supporting a likely role in fibrosis and cardiac remodeling [14]. Accordingly, we analyzed the expression levels of non-fibrillar collagen genes in LV tissue from patients with DCM and examined the impact of altered expression on cardiac remodeling parameters. We postulate that changes in the expression of non-fibrillar collagen genes are related to myocardial remodeling process, and therefore may affect pathogenesis in DCM patients.

## Methods

### Collection of samples

LV tissue samples were obtained from 23 explanted human hearts: 13 from patients with DCM and 10 from non-diseased controls (CNTs) for RNA-sequencing (RNA-seq) analysis. To improve the numerical base with a higher number of patients we increased the DCM samples up to 18 in RT-qPCR validation and up to 28 in protein analysis. The clinical history, ECG, Doppler echocardiography, hemodynamic studies, and coronary angiography data were available on patients. Non-ischemic DCM was diagnosed when patients had intact coronary arteries on coronary angiography, and LV systolic dysfunction (ejection fraction [EF], <40%) with a dilated non-hypertrophic left ventricle (LV end-diastolic diameter >55mm). Patients with primary valvular disease were excluded from the study. Patients were classified according to the New York Heart Association (NYHA) functional criteria and were receiving medical treatment according to the guidelines of the European Society of Cardiology [15].

All controls had normal LV function (EF >50%), as determined by Doppler echocardiography, and no history of cardiac disease. The CNT samples were obtained from non-diseased donor hearts that had been rejected for cardiac transplantation owing to size or blood type incompatibility, and due to the impossibility of finding a new recipient during the set period for transplant program. For these donors, the cause of death was either cerebrovascular events or motor vehicle accidents.

Access to operating rooms during interventions was allowed, reducing the time between reception of the sample and storage, and allowing the acquisition of high-quality samples, as evidenced by the values of the RNA integrity numbers (RIN; ≥9). Tissue samples were chosen from near the apex of the left ventricle in each procedure, were maintained in 0.9% NaCl, and were preserved at 4°C for a maximum of 6 hours after the loss of coronary circulation. Samples were stored at -80°C until use.

This study was approved by the Ethics Committee (Biomedical Investigation Ethics Committee of La Fe University Hospital of Valencia, Spain). Signed informed consent was obtained from each patient and control donors relatives prior to tissue collection. The investigation was conducted in accordance with the guidelines of the Declaration of Helsinki [16].

### RNA extraction and RNA sequencing analysis

The description of RNA isolation and RNA-seq procedure and analysis, are extensively described by Tarazón et al [17]. For Principal Component Analysis (PCA) we used ade4 R library [18], with the expression normalized counts of *COL4A5*, *COL8A1*, *COL9A1*, *COL16A1*, *COL21A1*, and *COL23A1* genes. For visualization, “gplots” R library was employed. The data described in this publication have been deposited in the NCBI Gene Expression Omnibus (GEO) and can be retrieved using the GEO Series accession number GSE55296 (http://www.ncbi.nlm.nih.gov/geo/query/acc.cgi?acc=GSE55296).

### RT-qPCR analysis

Reverse transcription was performed using 1 μg of total RNA and M-MLV enzyme (Invitrogen, UK) according to the manufacturer’s protocol. RT-qPCR was performed in duplicate in a ViiA7 Fast Real-Time PCR System according to the manufacturer’s instructions (Applied Biosystems; USA). The following TaqMan probes were obtained from Life Technologies: *COL8A1* (Hs00156669_m1), *COL16A1* (Hs00156876_m1), and the housekeeping genes *GAPDH* (Hs99999905_m1), *PGK1* (Hs99999906_m1), and *TFRC* (Hs00951083_m1) were used as endogenous controls. Relative gene expression levels were calculated using the 2^−ΔΔCT^ method [19].

### Protein analysis

Thirty milligrams of frozen LV samples were transferred into Lysing Matrix D tubes designed for use with the FastPrep-24 homogenizer (MP Biomedicals, USA) in total protein extraction buffer (2% SDS, 10 mM EDTA, 6 mM Tris-HCl, pH 7.4) with protease inhibitors (25 μg/mL aprotinin and 10 μg/mL leupeptin). The homogenates were centrifuged and the supernatants aliquoted. The protein content of aliquots was determined using Peterson’s modification of the micro Lowry method, using bovine serum albumin (BSA) as a standard [20].

For detection of COL8A1, TGF-β1, and MMP2, protein samples were separated by Bis-Tris electrophoresis on 4–12% polyacrylamide gels under reducing conditions. Tris-Acetate 3–8% electrophoresis under no-reducing conditions was used for COL16A1 detection. Polyacrylamide gels were transferred to a PVDF membrane using the iBlot Gel Transfer Device (Invitrogen, UK) for western blot analyses. The membranes were blocked overnight at 4°C with 1% BSA in Tris-buffer solution containing 0.05% Tween 20 and then were incubated for 2 h with the primary antibody in the same buffer. The primary detection antibodies used were anti-COL8A1 rabbit polyclonal (1:1000; ab-100988) obtained from Abcam (Cambridge, UK), anti- TGF-β1 mouse monoclonal (1:2000; ab-27969) obtained from Abcam (Cambridge, UK), anti-MMP2 mouse monoclonal (1:800; ab-80738) obtained from Abcam (Cambridge, UK), and anti-COL16A1 rabbit polyclonal (1:800; HPA027237) obtained from Sigma (St. Louis, USA); mouse monoclonal anti-GAPDH antibody (1:800; ab-9484) from Abcam was used as a loading control. Bands were visualized using an acid phosphatase conjugated secondary antibody and nitro blue tetrazolium/5-bromo-4-chloro-3-indolyl phosphate (NBT/BCIP, Sigma, St. Louis, USA) substrate system. Finally, the bands were digitalized using an image analyzer (DNR Bio-Imagining Systems, Israel) and quantified by the GelQuant Pro (v12.2) program.

### Statistical analysis

Data are presented as mean value ± standard deviation for continuous variables and as percentages for discrete variables. The Kolmogorov-Smirnov test was used to test for normal distribution of variables. Between-group comparisons of clinical characteristics were computed using the Student’s *t*-test (for continuous variables) or Fisher’s exact test (for discrete variables). Between-group comparisons of tissue mRNA and protein levels were performed using the Student’s *t*-test (for variables with a normal distribution) or the Mann-Whitney U test (for variables with a non-normal distribution). The mRNA levels of *COL9A1*, exhibit a non-normal distribution. Pearson’s correlation coefficient was used to examine associations between clinical parameters, and mRNA and protein levels (normally distributed variables); Spearman’s correlation coefficient was computed for non-normally-distributed variables. A *P*-value of < 0.05 was considered statistically significant. Genes with a fold change > 1.5 were considered as differentially expressed. All statistical analyses were performed using SPSS software v. 20 for Windows (IBM SPSS Inc., Chicago. IL, USA).

## Results

### Clinical Characteristics

We analyzed a total of 28 human explanted hearts from patients diagnosed with DCM, and CNT samples were acquired from 10 non-diseased donor hearts. DCM samples were homogenized by similar age and clinical characteristics, and were selected from patients with a larger number of clinical data available. DCM patients were mainly men in both RNA-seq and protein analysis (92% and 74%, respectively), with a mean age of 51 ± 11 years and 49 ± 13 years, respectively. Patients had an NYHA functional classification of III–IV and were previously diagnosed with significant comorbidities. The CNT group also mainly consisted of men (80%), with a similar mean age of 47 ± 16 years. Comorbidities and other echocardiographic parameters data of the control group were not available, in accordance with the Spanish Organic Law on Data Protection 15/1999. Patient clinical characteristics are summarized in Table 1.

**Table 1:** Clinical characteristics of patients with dilated cardiomyopathy.

	DCM (n = 13)	DCM (n = 18)		DCM (n = 28)	
	RNA-seq	RT-qPCR	*P*-value	Western blot	*P*-value
Age (years)	51±11	50±13	0.772	49±13	0.641
Gender male (%)	92	67	0.200	74	0.247
NYHA class	3.4±0.4	3.3±0.4	0.591	3.3±0.4	0.631
Prior smoking (%)	50	42	0.914	61	0.780
BMI (kg/m^2^)	27±5	25±5	0.254	25±6	0.355
Total cholesterol (mg/dL)	147±37	131±42	0.351	142±41	0.729
Prior hypertension (%)	17	17	0.517	31	0.508
Prior diabetes mellitus (%)	17	21	0.753	18	0.807
Hemoglobin (mg/mL)	13±3	12±3	0.281	13±3	0.962
Hematocrit (%)	39±8	36±8	0.291	40±7	0.822
Duration of disease (months)	75±68	63±60	0.674	71±59	0.863
***Echo-Doppler study***					
EF (%)	20±7	21±10	0.363	21±8	0.512
LVESD (mm)	71±12	67±12	0.217	65±12	0.175
LVEDD (mm)	80±11	74±12	0.187	74±11	0.182
LVMI (g/m^2^)	241±77	199±54	0.080	206±67	0.211

Data are showed as the mean value ± standard deviation. DCM, dilated cardiomyopathy; NYHA, New York Heart Association; BMI, body mass index; EF, ejection fraction; LVESD, left ventricular end-systolic diameter; LVEDD, left ventricular end-diastolic diameter; LVMI, left ventricular mass index.

### Gene expression analysis

**Fig 1:**
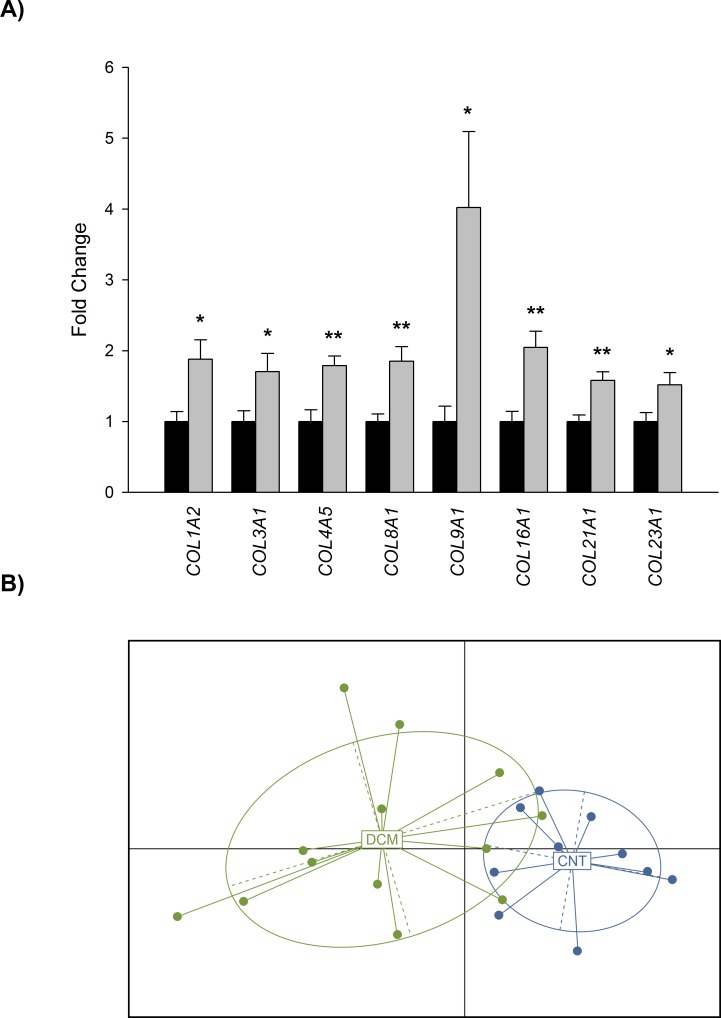
Altered expression levels of collagen genes in dilated hearts. (A) Bar graph comparing mRNA expression levels of collagen genes in dilated hearts (grey bars) *vs*. controls (dark bars). (B) Principal Component Analysis based on non-fibrillar collagen fold change values, shows a clear differentiation of the DCM and CNT groups. For RNA-seq analyses 13 LV samples from DCM patients were used. The values from the controls were set to 1. Bars display fold change (FC) ± standard error of the mean (SEM). Results were considered statistically significant at *P < 0.05 and **P < 0.01 *vs*. CNT.

Transcriptome-level differences between DCM and CNT samples were investigated by large-scale screening of 23 heart samples using RNA-seq technology. The number of reads obtained from the deep RNA-sequencing analysis was 36,688,217 of which 21,542,840 were mapped uniquely to the transcriptome. On comparing DCM and CNT samples, we found 1628 differentially expressed genes, of which 596 were upregulated (≥1.5-fold; P < 0.05 for all) and 1032 were downregulated (≥1.5-fold decrease; P < 0.05 for all) (These data have been deposited in the GEO; http://www.ncbi.nlm.nih.gov/geo/query/acc.cgi?acc=GSE55296). Focusing on collagen family, we found 8 differentially expressed collagen genes, all of them upregulated in the DCM samples (≥ 1.5-fold; *P* < 0.05 for all). Representative fold change values of expression levels of the collagen genes are shown in Fig 1A.

**Fig 2:**
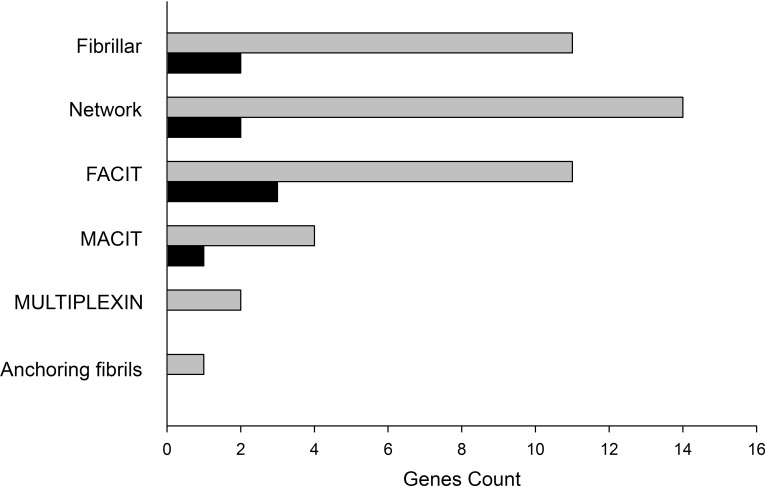
Classification of total collagen genes identified in RNA-seq analysis in dilated hearts. Bar graph shows total collagen genes identified for each class (grey bars) with their corresponding altered genes (dark bars). FACIT, fibril-associated collagens with interrupted triple helices; MACIT, membrane-associated collagens with interrupted triple helices; MULTIPLEXIN, multiple triple-helix domains and interruptions. Collagen genes have been ordered according to the *Shoulders*, *MD et*. *al*., classification [10].

We found significant differences in six non-fibrillar collagen genes belonging to Network (*COL4A5* and *COL8A1*), FACIT (*COL9A1*, *COL16A1*, and *COL21A1*), and MACIT (*COL23A1*) classes, representing 13.95% of total genes count against 4.65% of fibrillar genes found altered (Fig 2). We focused on non-fibrillar collagens and performed a PCA analysis to compare controls with these overexpressed genes in DCM. This analysis identified two divergent gene expression profiles that differentiate both DCM and CNT groups (Fig 1B).

In view of the contribution of collagens to cell adhesion, proliferation, migration, differentiation, apoptosis, fibrosis, and organization and morphology of tissues, we also investigated gene expression of known molecular markers of these processes. As shown in Table 2, we found differentially expressed markers in all categories, being all of them upregulated excluding IL-6 inflammation marker.

**Table 2:** Expressed mRNA levels of molecular markers of proliferation, hypertrophy, apoptosis, fibrosis and inflammation in DCM patients.

MOLECULAR MARKERS	Gene	FC ± SEM	*P* value
**PROLIFERATION&HYPERTROPHY**	***TGFβ1***	1.04 ± 0.10	NS
	***TGFβ2***	2.20 ± 0.44	< 0.05
	***TGFβ3***	1.32 ± 0.06	< 0.01
	***MMP2***	1.31 ± 0.09	< 0.05
	***MMP9***	0.34 ± 0.11	NS
	***ANP***	32 ± 13	< 0.0001
	***BNP***	24 ± 6	< 0.01
**APOPTOSIS**	***CASP3***	1.32 ± 0.11	< 0.05
	***CASP9***	1.33 ± 0.11	< 0.05
**FIBROSIS**	***COL1A2***	1.88 ± 0.27	< 0.05
	***COL3A1***	1.70 ± 0.26	< 0.05
**INFLAMMATION**	***TNFA***	0.56 ± 0.16	NS
	***IL6***	0.35 ± 0.09	< 0.01

Data are showed as the fold change (FC) value ± standard error of the mean (SEM).

### RT-qPCR and protein expression analyses

**Fig 3:**
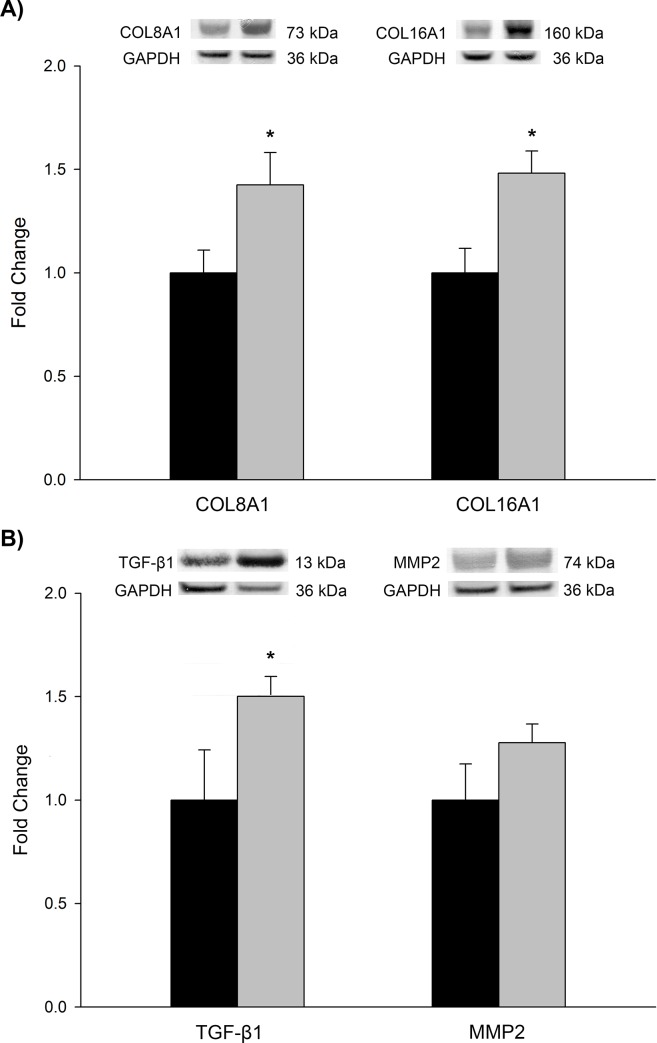
Protein expression levels of COL8A1, COL16A1, TGF-β1, and MMP2 in dilated cardiomyopathy. Bar graph comparing protein levels of non-fibrillar collagens COL8A1 and COL16A1 (A), and TGF-β1 and MMP2 (B) levels in dilated hearts (grey bars) *vs*. controls (dark bars). For western blot analyses 28 LV samples from DCM patients were used. The values from the controls were set to 1. Bars display fold change (FC) ± standard error of the mean (SEM). Results were considered statistically significant at *P < 0.05 *vs*. CNT.

We carried out RT-qPCR and western blot analyses focusing on *COL8A1*, related significantly with LV mass index, and on *COL16A1*, the new collagen related with ventricular dysfunction in ischemic cardiomyopathy (ICM) [14]. RT-qPCR results showed an identical trend in changes expression (2.02 fold, *P* < 0.01 and 2.45 fold, *P* < 0.001, respectively). To determine whether these results were translated into changes at the protein levels, western blot analyses were performed. Protein levels of *COL8A1* and *COL16A1* (142 ± 16 vs. 100 ± 11 arbitrary units [AU], *P* < 0.05; 148 ± 11 vs. 100 ± 12 AU, *P* < 0.05, respectively), were all in accordance with the previously measured mRNA levels (Fig 3). For the TGF-β1 role in the propagation of downstream intracellular signaling in a large variety of biological processes, its pleiotropic effects, and both TGF-β1 and MMP2 involvement in collagen turnover, we also investigated protein levels of these molecules. Although we found increased levels of both proteins in DCM samples compared to the CNTs (Fig 3), significantly differences were only obtained for TGF-β1 peptide (150 ± 10 vs. 100 ± 24 arbitrary units [AU], *P* < 0.05).

### Relationships between gene expression and remodeling parameters

**Fig 4:**
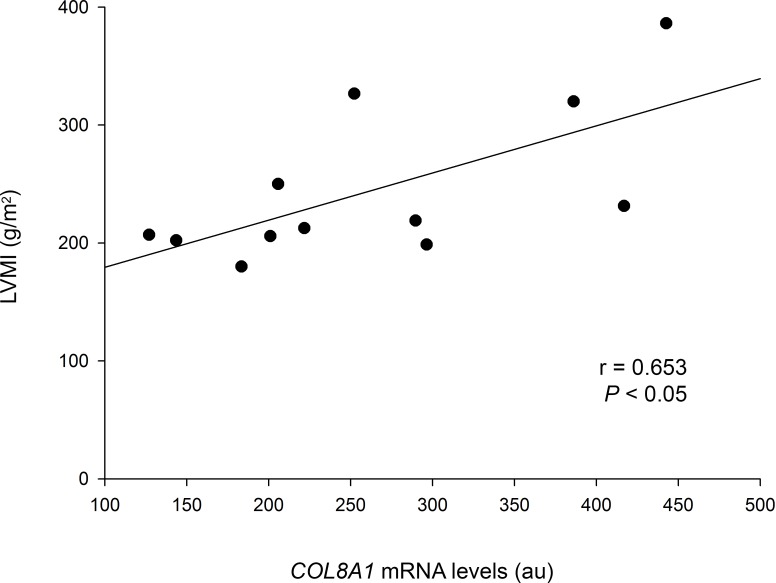
Relationship between collagen mRNA expression levels and remodeling parameters of DCM patients. Left ventricular mass index (LVMI) vs. *COL8A1* mRNA levels. Arbitrary units (au).

Next, we analyzed correlations between the mRNA expression levels of individual non-fibrillar collagen genes and remodeling parameters in the DCM group to investigate potential relationships and their clinical relevance. Interestingly, we observed a significant positive correlation between *COL8A1* gene and LV mass index (r = 0.653, *P* < 0.05) (Fig 4).

Furthermore, we found significant positive correlations between *COL8A1* and apoptosis, and fibrosis molecular markers (*COL8A1 vs*. *CASP3*, r = 0.735, *P* < 0.01; *COL8A1 vs*. *COL1A2*, r = 0.708, *P* < 0.01, respectively). We also observed additional relationships between COL8A1, COL16A1 and TGF-β1 levels (COL8A1 *vs*. COL16A1, r = 0.452, *P* < 0.05; COL8A1 *vs*. TGF-β1, r = 0.433, *P* < 0.05, COL16A1 *vs*. TGF-β1, r = 0.477, *P* < 0.05) (Table 3).

**Table 3:** Relationships between the differentially expressed non-fibrillar collagens COL8A1 and COL16A1, and proliferation, apoptosis, and fibrosis molecular markers at mRNA and protein levels in DCM patients.

	Gene	COL8A1	COL16A1
**RNA-SEQUENCING**	***TGFβ1***	NS	NS
	***CASP3***	r = 0.735**	NS
	***COL1A2***	r = 0.708**	NS
	***COL4A5***	NS	r = 0.641*
	***COL9A1***	NS	r = 0.697**
	***COL21A1***	NS	r = 0.634*
**RT-qPCR**	***COL8A1***	r = 1***	r = 0.813***
	***COL16A1***	r = 0.813***	r = 1***
**WESTERN BLOT**	***TGFβ1***	r = 0.433*	r = 0.477*
	***COL8A1***	r = 1***	r = 0.452*
	***COL16A1***	r = 0.452*	r = 1***

*P < 0.05

**P < 0.01

***P < 0.001.

## Discussion

In DCM, remodeling is characterized by cardiomyocyte hypertrophy, cell proliferation and migration, fibrosis, and apoptotic and necrotic cell death. Increased fibrosis and collagen turnover within the myocardium lead to ventricular stiffness and weakening of structural linkages, which is accompanied by the progression of cardiac dilation and LV dysfunction [21,22].

Numerous investigations are being conducted to elucidate the roles that diverse and versatile collagen proteins may play in fibrosis and progression of cardiomyopathies. Our previous studies in ICM suggested novel possible roles for non-fibrillar collagen genes in the compensatory remodeling process, and consequently in ventricular dysfunction [14]. In consideration of the potential roles of these collagens in ICM, we attempted to identify altered expression patterns of these genes in patients with DCM, and to investigate how their expression might be related to ventricular dysfunction.

Type VIII collagen synthesis significantly increases after injury and during development of atherosclerosis, supporting cell migration, attachment and focal adhesion of vascular smooth muscle cells (SMCs) [23,24]. In cell migration, cellular linking and release from the matrix occurs. This process is coordinated by matrix metalloproteinases that degrade ECM components, allowing cell translocation [25]. Type VIII collagen is also able to stimulate MMP-2 and MMP-9 synthesis in SMCs [23,26,27], and deletion of this collagen in mice results in impaired SMCs migration and decreased fibrillar type I collagen deposition [28].

On the other hand, up-regulation of type VIII collagen may induce apoptosis and hypertrophy in mesangial cells by modulating TGF-β1 [29]. TGF-β1 induces cell cycle inhibitors resulting in mesangial G1 phase arrest, which is associated with the development of hypertrophy [30–32]. Nevertheless, in the absence of type VIII collagen expression, TGF-β1 provokes cell cycle progression. Furthermore, in col8a1^-/-^/col8a2^-/-^ cells, a significantly reduced rate of apoptosis is observed in contrast to wild-type mesangial cells confirming the finding that in the absence of collagen VIII expression, TGF-β1 is able to induce cell survival pathways [29]. This effect of type VIII collagen inducing apoptosis has also been described in Bovine Aortic Endothelial cells [33].

Fibril-associated collagen XVI has been identified as a possible promotor of inflammatory process and fibrotic responses. It has been proposed that collagen XVI may promote formation and maturation of focal adhesion contacts on myofibroblasts, thus increasing pathological maintenance of these cells at the inflammation site, promoting fibrotic responses in the tissue and prolonging disturbances of cellular and ECM homeostasis [34].

In this study, using the reliable RNA-seq technique we identified upregulated mRNA levels of both *COL8A1* and *COL16A1* genes. The differential mRNA expression levels of these genes were validated by RT-qPCR. In addition, western blot analysis demonstrated correspondingly increased protein levels, reinforcing their relevance in this disease. We also found increased levels of both TGF-β1 and MMP2, being TGF-β1 significantly upregulated and related to both COL8A1 and COL16A1 protein levels, supporting the potential role of TGF-β1 in modulating non-fibrillar collagens response. In addition, we identified positive correlations between *COL8A1*, and apoptosis and fibrosis molecular markers, sustaining its potential involvement in these processes. Moreover, we found new myocardial non-fibrillar collagen genes that were previous not known to be overexpressed in DCM patients, such as *COL4A5*, *COL9A1*, *COL21A1* and *COL23A1*.

Collagen XXIII, is a transmembrane collagen that exists as a full-length membrane-anchored protein or as a soluble ectodomain. Proteolytic processing of this protein has been implicated in the activation of growth factors and protease cascades and has been related to a mechanism of environmental adaption after injury by inducing collagen XXIII ectodomain cleavage and further migration [35,36].

Similarly to collagen XXIII, type XXI collagen has not been reported in HF. Collagen XXI belongs to the FACIT class, which includes members such as collagens IX, XII, XIV and XVI, so it may associate with fibrillar collagens, such as type I and III, and have a role in their organization [10,37]. Type IV collagen is known to participate in fibrosis development in DCM hearts, probably inducing myofibroblast transdifferentiation. [38,39].

Overall, we suggest that upregulated types VIII and XXIII collagens may facilitate the migration of cardiac cells by stimulating the synthesis and activity of MMPs that detach cells from the matrix, and may lead to weakening structural cross-linking and augmented ventricular compliance. As mentioned above, upregulation of type VIII collagen is associated with increased apoptosis and hypertrophy. Interestingly, we observed that *COL8A1* mRNA levels were positively correlated with LV mass index (r = 0.653, *P* < 0.05). We postulate that collagen VIII could induce apoptosis and hypertrophy of cardiomyocytes in DCM through TGF-β1 modulation. Along with type VIII collagen, types IV and XVI collagens may be adding to the differentiation of myofibroblasts and their retention in the area of inflammation. Collagens IX and XXI, may additionally participate in the fibrotic process assisting the organization of fibrillar collagens. Positive correlations between *COL4A5*, *COL9A1*, *COL16A1*, and *COL21A1* observed, would be in accordance with a jointly action of non-fibrillar collagens. Thus, we hypothesize that the joint overexpression of a number of non-fibrillar collagen genes may promote pathological remodeling by inducing apoptosis and hypertrophy of cardiomyocytes, by adding to differentiation of myofibroblasts, and by assisting the organization of fibrillar collagens. Therefore, our findings propose new perspectives for DCM pathogenesis and provide new targets for further investigations to elucidate the role of these non-fibrillar collagens in modulating cardiac dilation, mass increase, fibrosis, remodeling and LV dysfunction.

### Study limitations

A common limitation of studies that examine cardiac tissues from end-stage HF patients is the presence of extensive variability in both disease etiology and treatment. The patients who participated in this study were pharmacologically treated, and some therapies might influence our results. Furthermore, tissue samples were taken from transmural left ventricle apex; therefore, our findings cannot be generalized to all layers and regions of the left ventricle.

## Conclusions

In this study, we used the RNA-seq technique to compare collagen gene expression levels in DCM and non-diseased cardiac tissue samples. Among the non-fibrillar collagen genes, we found mRNA and protein levels of *COL8A1* and *COL16A1* significantly increased in DCM samples. We also identified TGF-β1 significantly upregulated and related to both COL8A1 and COL16A1. Upregulated *COL4A5*, *COL9A1*, *COL21A1*, and *COL23A1* genes, encode collagens that have not previously been reported to be related to dilated cardiomyopathy. Of note, the overexpressed levels of *COL8A1* were significantly associated with pathological remodeling parameters, specifically with an increased mass index. These results suggest additional targets for future studies of the complex remodeling process that occurs in DCM, and could lead to the development of new therapeutic alternatives.
